# Synergist response of the Peach fruit fly, *Bactrocera zonata* (Saunders) to some ammonium based proteinaceous food bait attractants

**DOI:** 10.1186/s40850-023-00178-5

**Published:** 2023-09-04

**Authors:** Muhammad Hasnain, Shafqat Saeed, Unsar Naeem Ullah, Sami Ullah, Syed Muhammad Zaka

**Affiliations:** 1 Institute of Plan Protection, Faculty of Agriculture and Environmental Sciences, MNS University of Agriculture, Multan, Punjab Pakistan; 2 Department of Horticulture, Faculty of Agriculture and Environmental Sciences, MNS University of Agriculture, Multan, Punjab Pakistan; 3 Department of Entomology, Faculty of Agriculture and Environmental Sciences, Bahauddin Zakariya University, Multan, Punjab Pakistan

**Keywords:** *Bactrocera zonata*, Y-shape olfactometer, Food attractants, Ammonium compounds

## Abstract

**Background:**

Under the family Tephritidae, *Bactrocera zonata* (Saunders) is a serious pest, attacking fruits and vegetables causing large quantitative and qualitative damages throughout the world. Fruit flies require proteinaceous food for sexual maturation and egg development. Therefore, food bait attractants are frequently utilized for fruit fly detection, monitoring, mass trapping, and control. Using a Y-shape olfactometer (behavioral tests), we selected the best synthetic proteinaceous food bait attractants to volatiles identified by fruit fly antennae. The responses of *B. zonata* adults, male and female, to some ammonium compounds (ammonium acetate (AA), trimethylamine (TMA), and putrescine) that were mixed with certain food attractants were evaluated under laboratory conditions. Using flies ranging in age from 5 to 30 days, possible mixtures were discovered that might be useful in developing fruit fly attractants for both males and females. So, four base baits were developed by mixing protein hydrolysate with jaggery, papaya powder, kachri powder, potassium hydroxide (KOH), and guava pulp. Finally, thirty-two (32) synthetic blends were developed when the above four base baits were mixed with synthetic attractants.

**Results:**

The olfactometer bioassay indicated that protein hydrolysate and jaggery-based baits were effective in attracting both male and female flies throughout their adult lives when combined with AA + putrescine (Bait 6) and AA + TMA + putrescine (Bait 8). Similarly, protein hydrolysate + guava pulp-based baits combined with AA + putrescine (Bait 6) and AA + TMA + putrescine (Bait 8) was effective in attracting both male and female flies from 5 to 30 days of age. The pH of all 32 synthetic blends was measured and ranged from 4.77 to 11.35.

**Conclusions:**

According to our observation, the variation in pH may be due to differences in chemical composition between the attractants and food constituents. The pH of protein bait attractants may be an important factor in the attraction efficiency of *B. zonata* males and females.

## Background

The Peach fruit fly, *B. zonata* (Saunders) (Diptera: Tephritidae) is the most economically, dominant and serious pest of vegetables and fruits worldwide [1–3]. It is widely distributed in several countries including Pakistan, India, Sri Lanka, Nepal, Burma, China, Thailand, Egypt, and Vietnam [2–4]. It is considered highly invasive due to highly fecundity, fertility rate and great dispersal ability from one region to other region or from one country to other country through trade and export or import of infested fruits and vegetable so it is to be consider a key quarantine pest by several countries [5]. Tephritid fruit flies are polyphagous, attacking a variety of fruit and vegetable species, including mango, guava, citrus, peach, fig, apple, apricot, and tomato, as well as pepper and egg plants as secondary hosts [6, 7]. Losses due to favorable environmental conditions and crop sensitivity can range from 30 to 100% [8, 9]. Various types of conventional eradication techniques for the control of fruit fly being used are fruit bagging, chemical insecticides spraying, sex pheromone trap (methyl eugenol), Sterile Insect Technique (SIT), predator, parasitoid and Entomopathogenic fungi which affect the fruit fly adults to avoid fruit fly loses in the world [10]. Chemical control fenthion, diptrex and malathion used before mango ripening but control has not been sustainable [11–13]. This might lead to development of insecticides resistance, pest resurgence, environmental damage, maximum residual level and health hazard residues. Control of fruit flies is so difficult in many countries of the world because of systemic and broad-spectrum insecticides are unavailable or removed from the market [14]. Another drawback for the use of chemical application is that 3^rd^ instar larvae leave rotten fruits and drop to pupate in the ground soil, so both larvae in fruits and pupae in soils are well protected from insecticides surface application [15].

Male Annihilation Techniques (MAT) are commonly utilized against several fruit fly species for monitoring, mass trapping, suppression, and mating disruption using methyl eugenol, med lure, and cue-lure, among other things. The biggest drawback is that only males are attracted and captured [16]. Because of the enormous fly populations sustained by host plants available all year, and the geographic isolation of the release zone, suppression using SIT is not viable [17]. Incredible numbers of irradiated flies (Billions per week) are needed for SIT that is costly eradication program [18].

So, an alternate approach that is most efficient, cost-effective, and environmentally eco-friendly is the bait application technique, which is a proteinaceous food bait used for the attraction, detection, and control of both sexes (male and female) of *B. zonata*. Adult tephritid fruit flies, particularly females, require proteinaceous food material for sexual development and the maturation of gonads [19]. Several different types of synthetic food baits now a day have been developed and are being used as female attractants [20]. Early food bait attractants included fermented sugar, yeast, molasses, and protein hydrolysate. The protein hydrolysate fragrance is the most appealing to both sexes. As a result, synthetic food attractants (ammonium acetate (AA), trimethylamine (TMA), and putrescine (1,4-diaminobutane, Pu) mix with protein hydrolysate to maximize the attractiveness of male and female responses. These baits include a volatile chemical in the form of ammonia, which is the principal attractant for tephritid fruit flies [21]. Baits used commercially with different formulation ammonium bicarbonate and ammonium carbonate are available for fruit fly traps [22]. These proteinaceous food baits decomposes slowly and release ammonia fumes, a powerful affected food attractant for all fruit flies [23]. Alternatively, pH level of proteinaceous food baits is associated with ammonia which has been played a fundamental role to attract and mass capturing of fruit flies of Tephritidae [6, 24, 25].

The advantages of food baits are that they attract both females and males of various species, they are an alternative for controlling and monitoring male annihilation, and they are a synthetic lure derived from food volatiles that are detected by both sexes and directly target species control by removing female populations. The current study was aimed to test the efficiency of certain local food-based ammonium attractant compounds with protein hydrolysate on both male and female *B. zonata* species using an olfactometer. Proteinaceous food baits emitted volatile scent, which are essential factors in the attractiveness of both male and female fruit flies, and are consequently employed as bait for catching female fruit flies in particular. Historically, liquid proteinaceous food baits with ammonium-based compounds have been used to catch a wide range of different fruit flies [26]. The antenna is the primary organ responsible for chemoreception and olfactory stimulation in insects [27]. Antennae serve as a link between odours in the environment and insect behaviour [28].

The purpose of this study was to evaluate the proteinaceous food baits with local available attractants and extent of pH degree effect on the attraction of the Peach fruit fly, *B. zonata*.

## Methods

### Insect

*B. zonata* were reared in the laboratory of Institute of Plant Protection, MNS-University of Agriculture, Multan, Pakistan. Infested fruits of *B. zonata* were collected from different mango (*Mangifera indica* L., Anacardiaceae) orchards near the vicinity of Multan and placed in the plastic cage was having sterilized moist sand at the bottom for pupae collection. After a week, the sand was sieved for pupae collecting and adult emergence in a separate wooden cage (60 length × 40 widths × 40 heights in cm) made with 12 length × 2 widths in cm wire meshing to ensure consistent temperature and ventilation. The rearing conditions were set at 65—75% RH, 25 ± 2 ºC, and a photoperiod of dark and light of 12:12 h.

Emerged adult flies were provided with 3:1 mixture of sugar and yeast with small amount of water. A plastic cup with a wet cotton wick placed within the cage was also offered as a supply of water [29]. For oviposition, flies were offered fresh banana in the cage. Adult flies laid eggs in the banana fruits, the infested fruits were collected after 3–4 days from the flies’ cages and put them in the plastic bowls was having moist soil at the bottom and cover it with muslin cloth. After eggs hatched, larval period took 10–11 days then undergo the pupation stage. After emergence of flies, artificial diet along with banana was provided after 2–3 days. After 20–22 days, the flies began to lay eggs. As a result, new and consistent age adult flies were accessible for experimentation. *B. zonata* was reared on the same standard procedures followed [30]. Prior to experiment both male and female were starved for 24 h to normalize the physiological state of *B. zonata* to be tested [31].

### Preparation of protein hydrolysate

Fish meat (1 kg) was divided into four equal parts i.e., 250 g and were put in four different reagent bottles (1 L) separately. Distilled water (250 ml) was added in each of the reagent bottle. After this, all four bottles were placed in dry oven (POL-EKO, APARATURA) at 110 $^\circ{\rm C}$ for 48 h to hydrolyze and to convert protein into amino acid. After 48 h, the material of the bottle was strained to remove all impurities followed by cooling at room temperature [32]. Each of the strained material were then added with four different types of constituents {papaya powder (20 gm) + Kachri powder (20 gm); KOH (30 gm); Jaggery (200 gm); guava pulp (200 gm)} separately, to prepare four different baits. Each of the bait was further mixed with three types of attractants i.e., ammonium acetate (AA) (40 gm), trimethylamine (TMA) (40 ml) and putrescine (Pu) (0.20 ml). These attractants were further mixed collectively in different combinations to check their synergistic and antagonistic effects.

### The olfactometer system

**Fig. 1:**
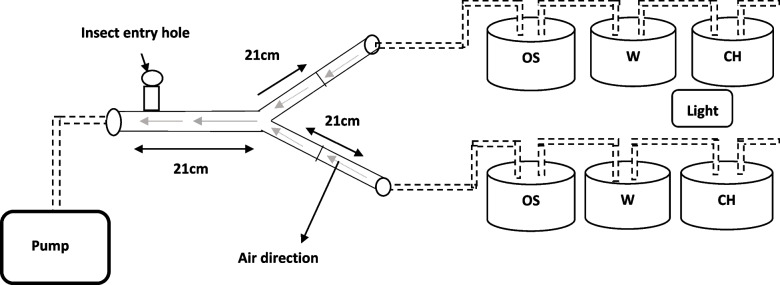
Schematic diagram of the Y-tube olfactometer (OS: odour source, W: water for humidity, CH: charcoal for air purification, L: light and dotted lines represent Teflon tubes)

The attractiveness of male and female towards synthetic food baits volatiles was assessed by using a Y-tube glass olfactometer [33, 34]. The olfactometer was made of a borosilicate glass having a main arm (21 cm long, 2 cm diameter) with two side arms (21 cm long, 2 cm diameter) shaped at a 45^0^ angle between the two arms. The Y-tube was placed in the center of the wooden box (36 cm × 38 cm × 57 cm) to disperse light more uniformly and to avoid disturbances by movements of the experimenter. Each side of arm was connected with three flasks (50 ml) having order source, distilled water and charcoal, respectively, and at the end air flow meter was adjusted to control the air flow. All flasks were inter-connected by Teflon tubing. To prevent flies from entering inside the flasks a sieve was placed at end of each arm. A halogen lamp illuminated the Y-junction of the olfactometer with 160 lx light intensity while all others lights were kept off (Fig. 1). Insects were released through main arms of the olfactometer. A suction pump was connected to the olfactometer and an air flow of 8-kpascal was maintained [34].

### Standardization testing conditions for the olfactometer

To check the working efficiency of the olfactometer, release five numbers of fruit flies (male or female) in the olfactometer when each arm is kept empty to check the movement within 2 min. In the second test, a similar number of fruit flies were released when one arm had protein hydrolysate and the other arm was kept empty. Count and compare the number of fruit flies (male or female) towards the baited arm other than that of the empty arm within 2 min with an 8-kpascal air flow.

### Bioassay analysis

All the prepared attractant baits were tested for their possible attraction or repulsion in y-tube olfactometer. Different combinations of treatments as mentioned in Table 1 were checked separately against male and female population of different ages (5, 10, 15, 20, 25 and 30 days old). Each olfactometer trial was replicated 5 times and each of the replication was consisted of 5 flies. All flies before olfactory test were given 24 h starvation stresses in order to enhance the choice process [35]. Two minutes time was given to adults for making choice among two arms. Five fruit flies were released inside the main arm of olfactometer by using a small size aspirator and observed their response within 2 min. One arm of the flask having baits (5 ml socked with cotton) and other arm of the flask was having cotton wick socked with water. The number of female and male flies moves towards the baited arm and water socked wick arms were counted during each bait tested. Activated by the volatile odour loaded air flow and additionally motivated by the light, flies walked towards the arms of the tube. If those flies did not enter inside any arm of the olfactometer after 2 min were classified as “no choice” and were discarded. The number of fruit flies went toward each upper end of the olfactometer was recorded. The positions of the odour’s sources were exchanged after two repeats to avoid bias by accidental asymmetry in the experimental setup. After every odour source tested, olfactometer was once clean with soap and 70% ethanol followed by distilled water and then dried.

**Table 1:** List of chemical composition of Jaggery based baits

Baits name	Chemical composition of baits	pH of baits
Bait 1	Protein hydrolysate + Jaggery	5.44
Bait 2	Protein hydrolysate + Jaggery + AA	6.03
Bait 3	Protein hydrolysate + Jaggery + TMA	4.77
Bait 4	Protein hydrolysate + Jaggery + Putrescine	6.72
Bait 5	Protein hydrolysate + Jaggery + AA + TMA	6.55
Bait 6	Protein hydrolysate + Jaggery + AA + Putrescine	6.23
Bait 7	Protein hydrolysate + Jaggery + TMA + Putrescine	5.47
Bait 8	Protein hydrolysate + Jaggery + AA + TMA + Putrescine	6.18

*AA* stands for ammonium acetate, *TMA* for trimethylamine

### Data analysis

For the laboratory analysis, a two-choice sample paired t-test (*P* = 0.05) was performed to compare the attractancy of male and female fruit fly. The data for olfaction trail was compared by using t-test at 95% confidence level, further all the date was converted into percentage basis for the easiest to elaborate the results. All the data were statistically using a software program (SAS, 2002).

### Estimating pH levels

Each as a fresh baits sample was taken 10 ml for the estimation of pH level of synthetic proteinaceous food baits analysis. By using High- Performance Bench Meter for Universal Applications “OHAUS Stater 5000 pH Bench Meter”.

## Results

### Response of female fruit flies towards jaggery based baits

**Fig. 2:**
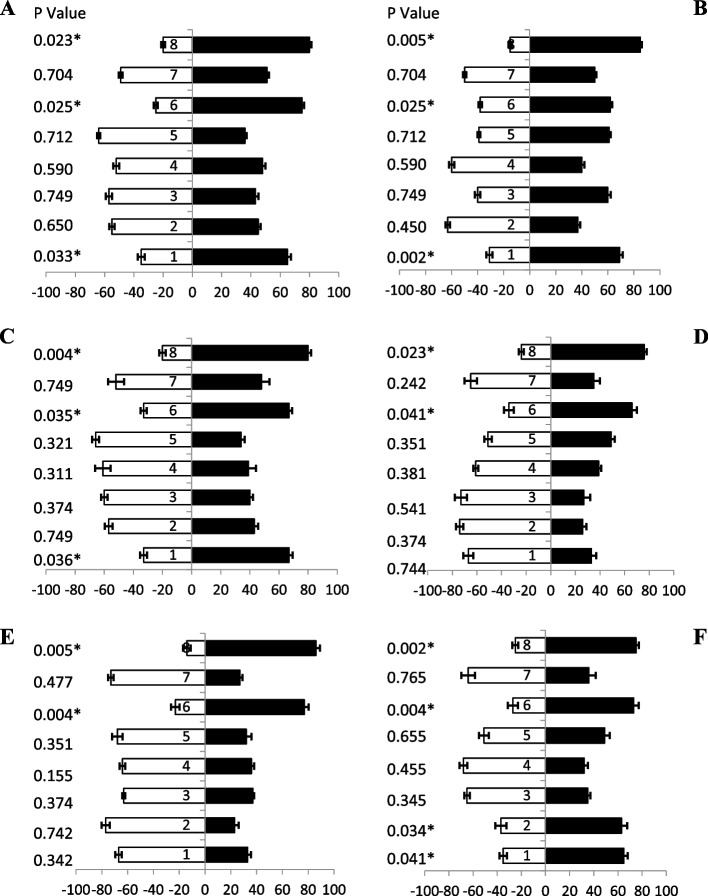
Behavioral response of female *B. zonata* towards different jaggery based food bait 1–8 of one arm and water socked cotton wick on the other arm of flask. Percentage responses after 5–30 days denoted by **A-F**, respectively. Black bars showed a choice and white bar for non-responses made by flies. *Indicates significant differences at *P* < 0.05, used t-test

When female’s fruit flies (of different ages) were tested against jaggery based protein hydrolysate baits (Table 1), a significant attraction was observed. Fresh fruit flies (5 day old) showed more attraction responses at different combinations of ammonium compounds towards baits attractant i.e., bait 1, bait 6 and, bait 8 with 65%, 75% and 80% attraction, respectively (Fig. 2A). Similarly, 10 days old flies also showed significant attraction towards baits with ammonium compounds attractants i.e., bait 8, 1, 6, 5 and, bait 3 with 85%, 69%, 62%, 61% and 60% attraction, respectively (Fig. 2B). Likewise, ammonium-based bait 8 showed highest attraction significantly (80%) by fruit flies (15 days old) as compared to other baits, while bait 6 and 1 showed same percentage of attraction 67% (Fig. 2C). Fruit flies (20 days old) responses maximum attraction towards baits 8 and bait 6 both having i.e., 76% and 66% attractant, respectively (Fig. 2D). Significantly, similar responses were observed (25 days old) toward baits 8 and baits 6 showed 86% and 77% attraction, respectively (Fig. 2E). Fruit flies (30 days old) highly volatile attractant response towards baits 8, 6,1 and 2 showed 75%, 73%, 65%, and 63%, respectively (Fig. 2F). These finding proved that female adults of all ages prefer the jaggery based bait with ammonium compounds for maximum attraction.

### Response of male fruit flies towards jaggery based baits

**Fig. 3:**
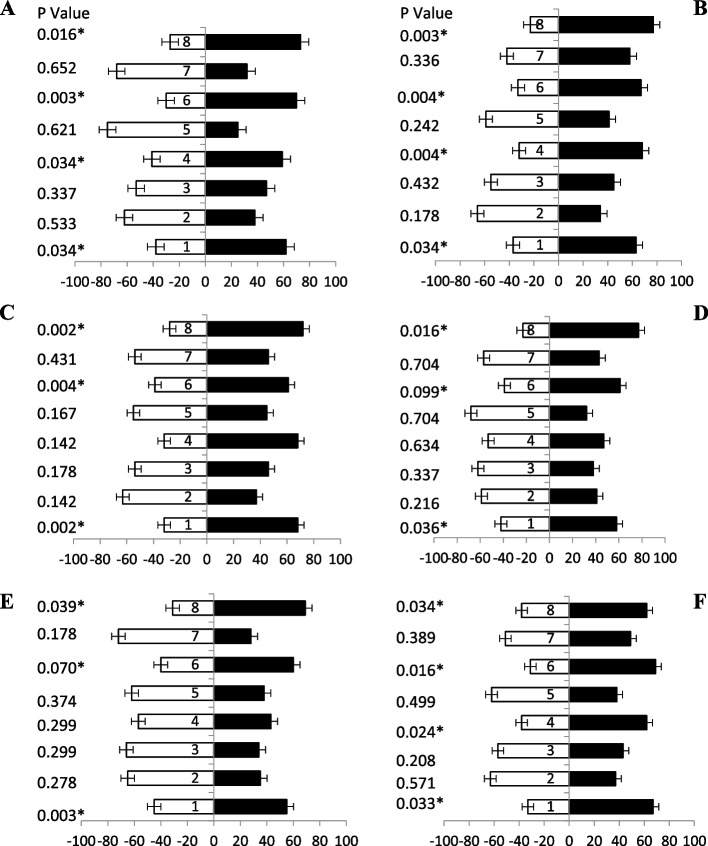
Behavioral response of male *B. zonata* towards different jaggery based food bait 1–8 of one arm and water socked cotton wick on the other arm of flask. Percentage responses after 5–30 days denoted by **A-F**, respectively. Black bars showed a choice and white bar for non-responses made by flies. *Indicates significant differences at *P* < 0.05, used t-test

Similarly, when different ages of male’s fruit flies were given jaggery based protein hydrolysate baits, a significant attraction was observed. Fruit flies (5 day old) data revealed more attraction response towards baits with ammonium-based attractant i.e., bait 8, bait 6, bait 1 and bait 4 having 73%, 70%, 62% and 59% attraction responses, respectively (Fig. 3A).

Data revealed after 10 days old flies also showed significant attraction towards baits having ammonium acetate attractant i.e., bait 8, bait 4, bait 6 and bait 1 having 77%, 68%, 67% and 63% attraction, respectively (Fig. 3B). Bait 8, bait 1, bait 4 and bait 6 contain tri-methylamine also show good attraction having 72%,68%,68% and 61% attractant, respectively (Fig. 3C). Likewise, fruit flies (20 days old) were response maximum attraction towards baits 8, bait 6 and bait 1 showed different degree of responses i.e., 77%, 61% and 58% attractant, respectively (Fig. 3D). Significantly more attraction towards baits having ammonium acetate attractant i.e., baits 8, baits 6, bait 4, and baits 1 showed 69%, 60%, 57% and 55% attraction towards fruit flies (25 days old), respectively (Fig. 3E). Volatile attraction responses of fruit flies (30 days old) were towards baits 6, bait 1, bait 4 and bait 8 also exhibited 69%, 67%,62% and 62% attractant, respectively (Fig. 3F). These results proved that male adults of all ages prefer the bait having ammonium salts with jaggery.

### Response of female fruit flies towards KOH based baits

**Fig. 4:**
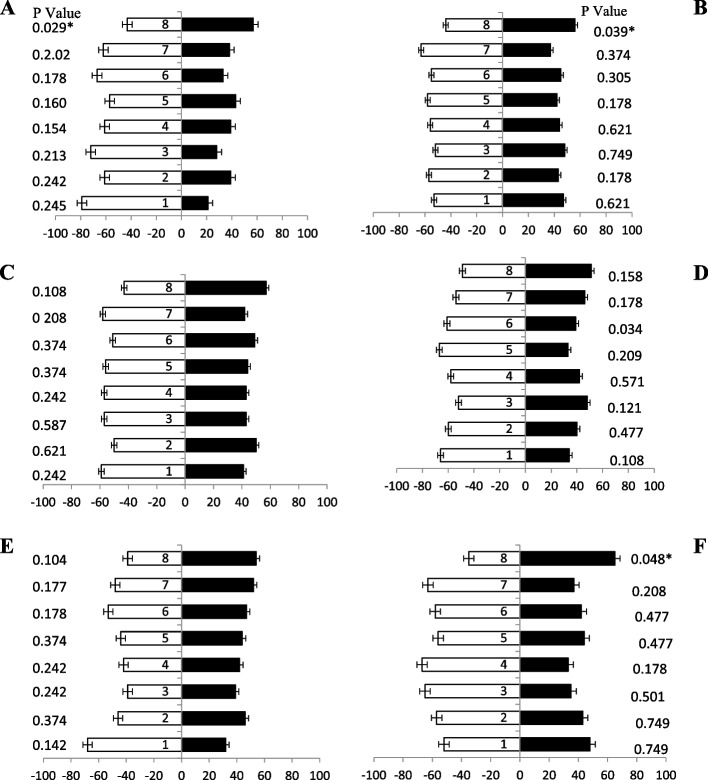
Behavioral response of female *B. zonata* towards different KOH based food bait 1–8 of one arm and water socked cotton wick on the other arm of flask. Percentage responses after 5–30 days denoted by **A-F**, respectively. Black bars showed a choice and white bar for non-responses made by flies. *Indicates significant differences at *P* < 0.05, used t-test

The response of different ages of fruit fliers were observed when KOH based protein hydrolysate baits tested. Maximum number of fruit flies’ attraction (5 days old) was observed (Fig. 4A) towards bait 8 only having 57% attractant. Likewise, fruit flies after 10 days old was attracted only bait 8 also having 56% attraction (Fig. 4B) others baits were not responding significantly. During 15 days old fruit flies was showed no significantly attracted towards any of the baits except bait 8 showed 57% attraction was observed (Fig. 4C). Twenty, twenty-five, and thirty-days old fruit flies showed not significantly attraction responses towards all the baits tested by olfactometer (Fig. 4D, E & F). These results showed that these baits having KOH respond only early adult ages having ammonium salts and its derivatives.

### Response of male fruit flies towards KOH based baits

**Fig. 5:**
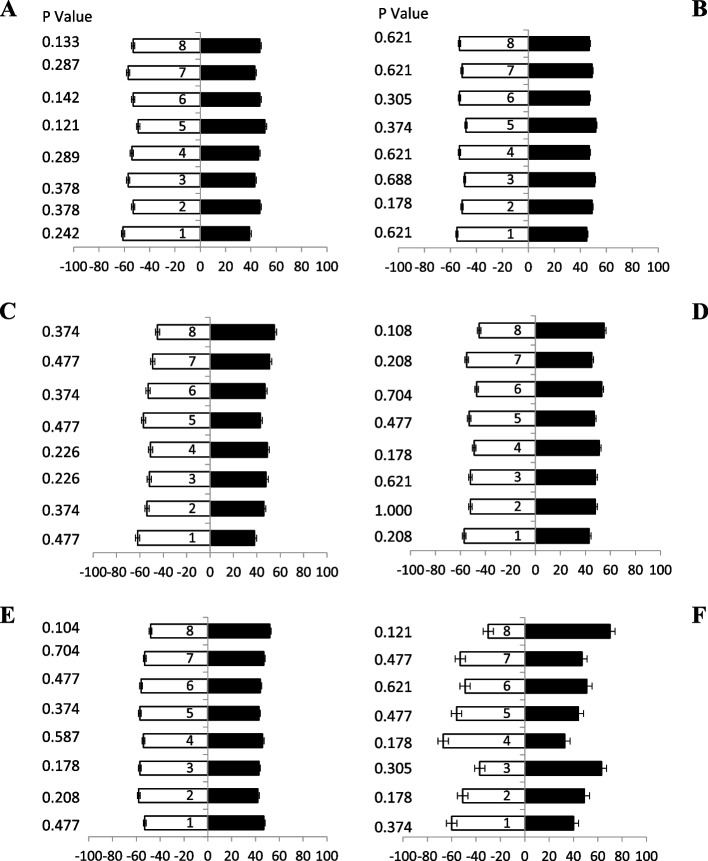
Behavioral response of male *B. zonata* towards different KOH based food bait 1–8 of one arm and water socked cotton wick on the other arm of flask. Percentage responses after 5–30 days denoted by **A-F**, respectively. Black bars showed a choice and white bar for non-responses made by flies. *Indicates significant differences at *P* < 0.05, used t-test

When KOH based protein hydrolysate were observed the response of fruit fliers of different ages were observed (Table 2). The fruit flies (5 days old) examined maximum attraction was observed (Fig. 5A) towards bait 5, followed by 8 and bait 3 both only having 51%, 47% and 43% attractant, respectively. Likewise, fruit flies (10, 15, 20 and 25 days old) were not significantly attracted towards all the baits (Figs. 5B, C, D & E). Maximum volatile attraction responses of fruit flies (30 days old) were towards baits 8 exhibited 70% irrespective to the others (Fig. 5F). These results indicated that baits having KOH based was not shown significant attraction towards food attractant.

**Table 2:** List of chemical composition of KOH based baits

Baits name	Chemical composition of baits	pH of baits
Bait 1	Protein hydrolysate + KOH	10.56
Bait 2	Protein hydrolysate + KOH + AA	8.70
Bait 3	Protein hydrolysate + KOH + TMA	11.31
Bait 4	Protein hydrolysate + KOH + Putrescine	8.49
Bait 5	Protein hydrolysate + KOH + AA + TMA	9.04
Bait 6	Protein hydrolysate + KOH + AA + Putrescine	11.07
Bait 7	Protein hydrolysate + KOH + TMA + Putrescine	8.77
Bait 8	Protein hydrolysate + KOH + AA + TMA + Putrescine	9.45

*AA* stands for ammonium acetate, *TMA* for trimethylamine

### Response of female fruit flies towards papaya powder + kachri based baits

**Fig. 6:**
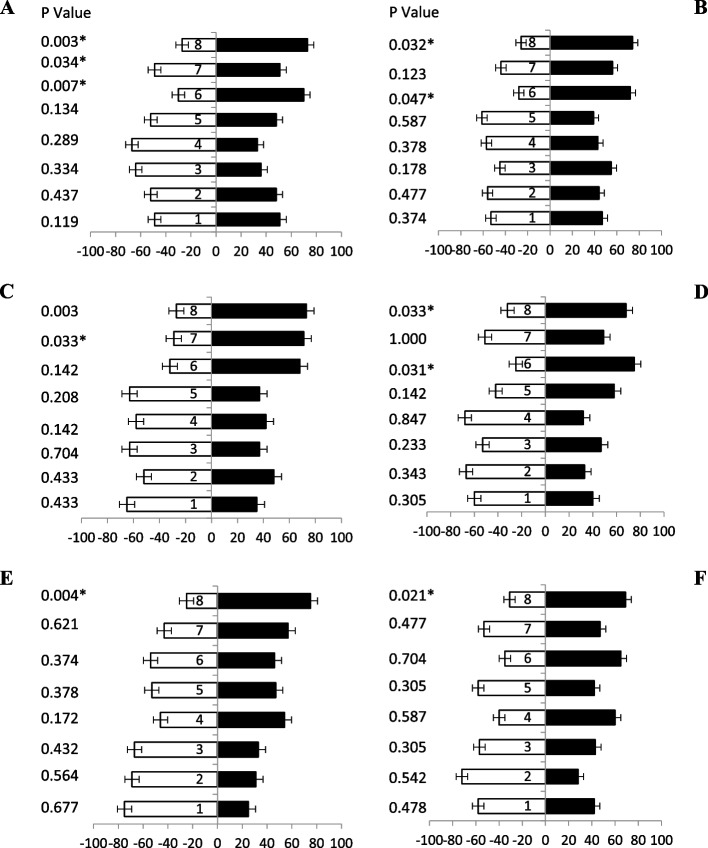
Behavioral response of female *B. zonata* towards different papaya + kachri powder-based food bait 1–8 of one arm and water socked cotton wick on the other arm of flask. Percentage responses after 5–30 days denoted by **A-F**, respectively. Black bars showed a choice and white bar for non-responses made by flies. *Indicates significant differences at *P* < 0.05, used t-test

The olfactometer study revealed that 5-day old fruit flies showed highly significant attraction 73% towards the bait 8 followed by baits 6 having 70% (Fig. 6A). All reaming baits showed relatively low significant effect (Table 3). After 10-day old fruit flies responded volatile odour towards the arm having food bait 8 and bait 6 was having 74% and 72% attraction (Fig. 6B), respectively. While other baits were not shown any significant attraction during the olfaction testing. Data revealed that after 15-day old fruit flies also responded (Fig. 6C) towards the bait 8, bait 7 and bait 6 having 73%, 71% and 68% attraction, respectively. Olfaction responses (20 days old) of fruit flies were only toward baits 6 and bait 8 having 75% and 68% responses, respectively. While all the other baits were not significantly responded towards odour source attraction (Fig. 6D). Volatile odour response after 25-day old fruit flies towards bait 8 was exposed maximum responses i.e., 75% only (Fig. 6E), among the others baits. Fruit flies (30 days old) were exhibited significantly highest odour response towards bait 8, followed by bait 6 and 4 i.e., 69%, 65% and 60% attraction, respectively (Fig. 6F).

**Table 3:** List of chemical composition of papaya powder + kachri powder-based baits

Baits name	Chemical composition of baits	pH of baits
Bait 1	Protein hydrolysate + papaya powder + kachri powder	7.56
Bait 2	Protein hydrolysate + papaya powder + kachri powder + AA	8.96
Bait 3	Protein hydrolysate + papaya powder + kachri powder + TMA	11.35
Bait 4	Protein hydrolysate + papaya powder + kachri powder + Pu	9.02
Bait 5	Protein hydrolysate + papaya powder + kachri powder + AA + TMA	10.28
Bait 6	Protein hydrolysate + papaya powder + kachri powder + AA + Pu	9.37
Bait 7	Protein hydrolysate + papaya powder + kachri powder + TMA + Pu	8.47
Bait 8	Protein hydrolysate + papaya powder + kachri powder + AA + TMA + Pu	6.89

*AA* stands for ammonium acetate, *TMA* for trimethylamine, *Pu *for putrescine

### Response of male fruit flies towards papaya powder + kachri based baits

**Fig. 7:**
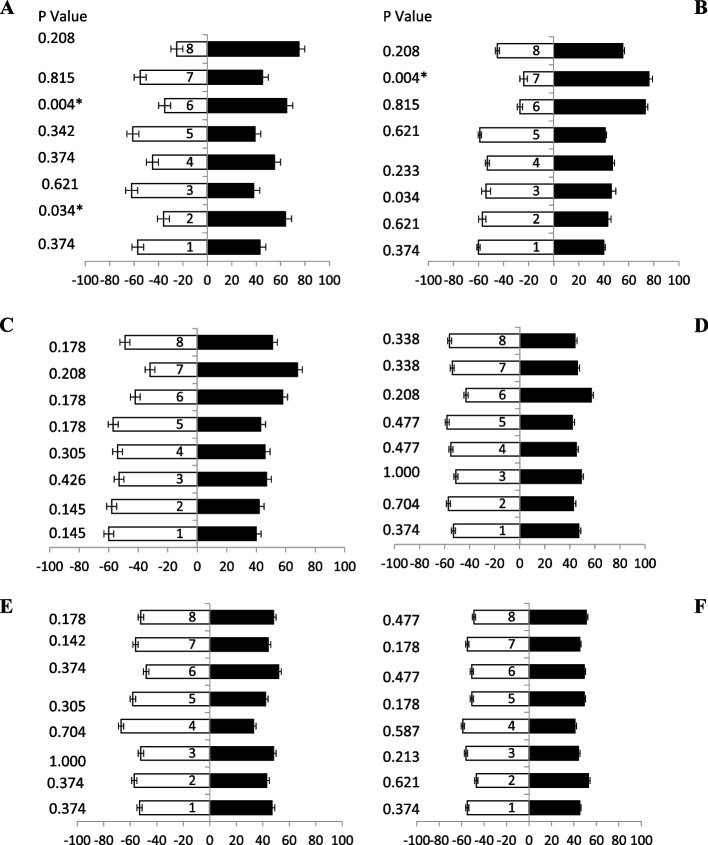
Behavioral response of male *B. zonata* towards different papaya + kachri powder-based food bait 1–8 of one arm and water socked cotton wick on the other arm of flask. Percentage responses after 5–30 days denoted by **A-F**, respectively. Black bars showed a choice and white bar for non-responses made by flies. *Indicates significant differences at *P* < 0.05, used t-test

Data revealed (Fig. 7A) that 5-day old fruit flies were significantly attracted towards bait 8, 6 and bait 2 having ammonium-based food attractants i.e., 75, 65 and 64% responses, respectively. Whereas other baits were not showing any response towards fruit flies. When 10-day old fruit flies (Fig. 7B) showed volatile attraction towards the bait 7 and bait 6 having 76% and 73% attractant, respectively. While other baits were not significantly shown any attraction during the olfaction testing. Data revealed that after 15-day old fruit flies also responded towards the bait 7 and 6 having 68 and 58% attractant, respectively (Fig. 7C). A relatively low significant olfaction response (20, 25, and 30 days old) of fruit flies towards any attractant baits was observed (Fig. 7D, E & F).

### Responses of female fruit flies towards guava pulp-based baits

**Fig. 8:**
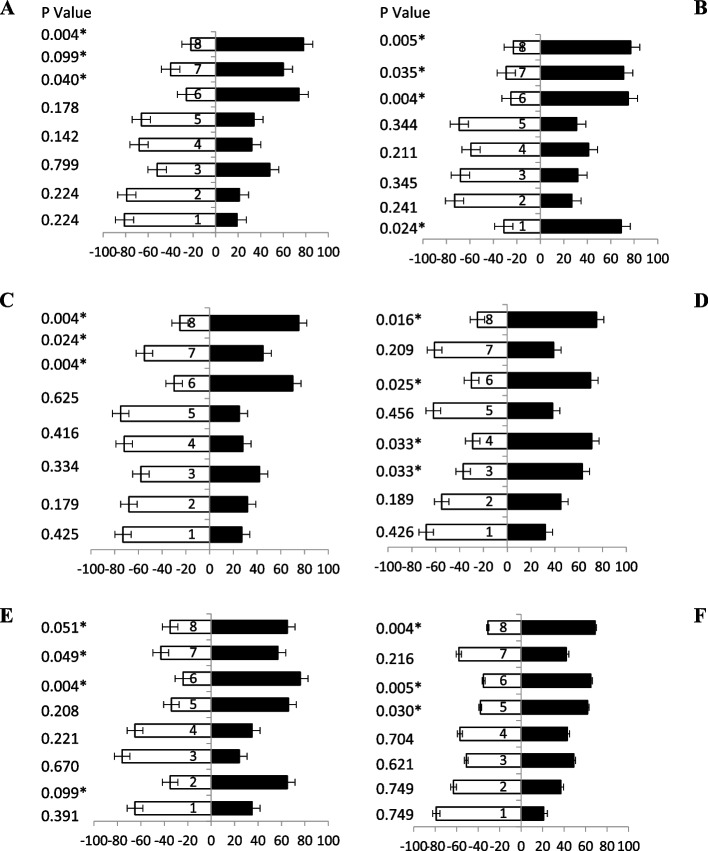
Behavioral response of female *B. zonata* towards different guava pulp based food bait 1–8 of one arm and water socked cotton wick on the other arm of flask. Percentage responses after 5–30 days denoted by **A**-**F**, respectively. Black bars showed a choice and white bar for non-responses made by flies. *Indicates significant differences at *P* < 0.05, used t-test

During olfactometer testing, 5-day old fruit flies had indicated a high attraction (Fig. 8A) towards bait 8, bait 6, and bait 7 having 78, 74, and 60% responses, respectively. However, the response of flies towards other baits were not remarkable. Fruit flies (10 day old) olfaction attraction of odour volatiles was a response towards bait 8, bait 6, bait 7 and bait 1 having 77, 75, 71 and 69% attractant (Fig. 8B), respectively. The maximum volatile attraction of fruit flies was (15 days old) revealed towards the bait 8 and bait 6 having 75 and 70% while the minimum attraction was observed towards the others baits having less than 50% attraction (Fig. 8C). While the intermediate attraction was observed bait 8, bait 4, bait 6 and bait 2 having 73, 72, 70 and 68% attractant, respectively. After 20-day old fruit flies indicated maximum attraction (Fig. 8D) towards baits 8 with 75% and followed by bait 6, bait 4 and bait 3 with 70, 71 and 63% attractant, respectively. Similarly, 25-day old fruit flies (Fig. 8E) responded significantly towards volatiles bait 6, bait 5, bait 8, and bait 2 i.e., 76, 66, 65 and 65% attractant, respectively. The volatile responses (Fig. 8F) after 30 days of fruit flies were towards bait 8 followed by bait 6 and baits 5 having 69, 65 and 62% attractant, individually (Table 4).

**Table 4:** List of chemical composition of guava pulp-based baits

Baits name	Chemical composition of baits	pH of baits
Bait 1	Protein hydrolysate + Guava pulp	6.39
Bait 2	Protein hydrolysate + Guava pulp + AA	6.35
Bait 3	Protein hydrolysate + Guava pulp + TMA	7.66
Bait 4	Protein hydrolysate + Guava pulp + Pu	8.49
Bait 5	Protein hydrolysate + Guava pulp + AA + TMA	5.39
Bait 6	Protein hydrolysate + Guava pulp + AA + Pu	6.46
Bait 7	Protein hydrolysate + Guava pulp + TMA + Pu	7.20
Bait 8	Protein hydrolysate + Guava pulp + AA + TMA + Pu	6.64

*AA* stands for ammonium acetate, *TMA* for trimethylamine, *Pu* for putrescine

### Response of male fruit flies towards guava pulp-based baits

**Fig. 9:**
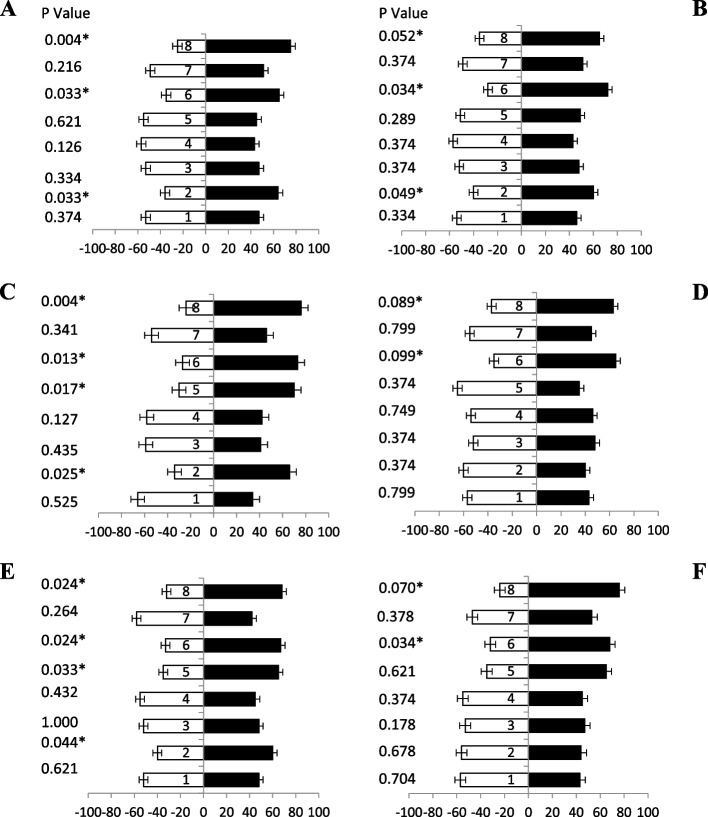
Behavioral response of male *B. zonata* towards different guava pulp-based food bait 1–8 of one arm and water socked cotton wick on the other arm of flask. Percentage responses after 5–30 days denoted by **A**-**F**, respectively. Black bars showed a choice and white bar for non-responses made by flies. *Indicates significant differences at *P* < 0.05, used t-test

A significant attraction was observed when male fruit flies (of different ages) were given guava pulp-based protein hydrolysate baits. Fresh fruit flies (5 day old) showed more attraction response (Fig. 9A) towards baits 8, bait 6 and bait 2 having 75, 65 and 64% attraction, respectively. Significantly, 10 days old flies also showed good attraction towards baits having ammonium acetate attractant i.e., bait 6, bait 8 and bait 2 having 72, 65 and 60% attractions (Fig. 9B), respectively. Likewise, these three food-based attractants showed good attraction towards fruit flies (15 days old) baits, i.e., bait 8, bait 6, bait 5 and bait 2 having 76, 73, 70 and 66% attractant (Fig. 9C), respectively. Fruit flies (20 days old) responded with maximum attraction towards bait 6 and bait 8 having ammonium compounds in combination, i.e., 65 and 63% responses (Fig. 9D). Significantly, baits 8, 6, bait 5 and 2 showed 68, 67, 65 and 60% attraction towards (Fig. 9E) fruit flies (25 days old), respectively. Also, fruit flies (30 days old) were attractant volatile order response towards baits 8, bait 6 and bait 5 also having ammonium acetate and putrescine showed 76, 68 and 65% attraction (Fig. 9F), respectively. These results proved that male adults of all ages prefer the bait having ammonium acetate-based food attractant.

### Ammonia and pH degree on the attractiveness of *B. zonata*

There was different variation in baits pH degree levels due to the different chemical combination i.e., alkaline and acidic in nature. The pH values of the jaggery-based synthetic proteinaceous lures with different combinations showed different attraction levels. In jaggary-based attractants, the highest number of responses both sexes attracted towards the bait 1, bait 6 and bait 8 having pH values level at 5.44, 6.23 and, 6.18, respectively.

In the case of papaya powder + kachri powder-based attractant, the result indicated that both males and females of fruit flies attract significantly less responses were showed towards the baits due to fluctuation of pH degree levels during olfactory analysis in the laboratory. Among males and females, males showed non-significant responses to the baits due to alkaline pH (ranged 7.56 to 11.35) in two-choice laboratory bioassays.

So, enticement 1, 2, 3, 4, 5, 6, 7, and 8 having pH degree values 7.56, 8.96, 11.35, 9.02, 10.28, 9.37, 8.47, and 6.89, respectively. KOH-based baits the response of males and females was non-significant due to KOH increasing the more pH alkalinity level than jaggery, papaya powder + kachri powder and guava pulp-based baits attractants. Baits 1, 2, 3, 4, 5, 6, 7, and 8 having pH degree values were 10.56, 8.70, 11.31, 8.49, 9.04, 11.07, 8.77, and 9.45, respectively. This result indicated that increased the alkalinity of the solutions ultimately increased the pH level and reduced the attraction of the fruit flies due to reduced decomposed protein. Guava pulp-based baits showed a significant result in attracting *B. zonata* fruit flies. Data revealed that bait 6 and 8 having pH degree levels, i.e., 6.46 and 6.46, respectively, attracted higher females. Male flies showed a higher attraction towards baits 2, having pH 6.35.

## Discussion

In this study, our aim was to identify key mixtures that attract both male and female *B. zonata* fruit flies towards olfactory odour stimuli, with the goal of developing synthetic proteinaceous food baits as attractants. These baits contain protein hydrolysate and other constituents of attractants such as jaggery, papaya powder + kachri powder, guava pulp, and potassium hydroxide (KOH), with the addition of ammonium acetate, tri-methylamine, and putrescine to enhance their attraction efficiency. A total of 32 food based olfactory attractants were screened out against *B. zonata*, both male and especially female, that cause maximum losses in the field. The result obtained in the present study revealed that adults (both male and female) of *B. zonata* have positive response towards the jaggery and guava pulp-based baits with different combination of food attractants (ammonium acetate, trimethylamine and putrescine) and proved to be significant preferable attractants of fruit flies throughout their life span of different days (5, 10, 15, 20, 25 to 30 days).

The result obtained in the present study revealed that both male and female of *B. zonata* showed highly significant attraction towards the baits having ammonium acetate food attractants. Similar results were found by many researchers who cited that *B. zonata* was attracted to different food attractant having ammonium compounds and the females of Peach fruit fly and Mediterranean fruit fly were more attracted to food attractants than males [6, 36–38]. The current experimental finding is in accordance with studies on the attraction of the Mexican fruit flies *Anastrepha ludens* (Loew) mentioned that ammonium acetate release ammonia and acetic acid that is useful for the attraction [39].

The results revealed that significant attraction of male and female *B. zonata* fruit flies were observed in jaggery with ammonium acetate-based baits when pH level ranged from 5.44 to 6.72. These results are consistent with the previous study that showed a pH level ranged from 5.5 to 8.5 had a 79.25% positive response from total males and females. On the other hand, when pH level was between 3.73 and 4.43, there was less or non-significant attraction to *Ceratitis capitata* [40]. Additionally, when the pH levels were lower than 5.5 or higher than 8.5, there were few numbers of attracted *Bactrocera* flies. The attraction of fruit flies to the baits was affected by pH becoming more acidic or alkaline [26].

During tunnel bioassays, the release rate of ammonia plays a very important role in the attraction of insects towards ammonium-based baits. A low release rate attracts more flies than high ammonia, which can actually be repellent [41]. In contrast, a low release of ammonia is associated with low attractiveness of proteinaceous baits, whereas higher gaseous ammonia released due to fertilizer (ammonium nitrate) and manure cause higher attractiveness to female *C. capitata* under laboratory condition [42].

*Bactrocera* tau (Walker) was found to be most attracted to baits containing jaggery mixed with ammonium acetate + ethyl methyl ketone, and ammonium acetate + water + sugar + ethyl methyl ketone was found to be the best food attractant for *Bactrocera* fruit flies. [43]. These results confirm the experimental finding that protein hydrolysate + jaggery solutions was found to be attractive to both sexes of several species of insect pests [44]. This discovery provides the potential to develop more efficacious and dependable baits for use in monitoring fruit fly activity in fruits orchards and other vegetables crops. The liquid hydrolyzed proteinaceous bait was found to attract more female oriental fruit flies in guava orchard as compared to several ammonia based olfactory lures [45].

Ammonium carbonate and ammonium acetate attract maximum number of *B. zonata* and *C. capitata* as compared to others ammonium compounds. Such differences due to different response among species and different physiological state i.e., age, sexually matured fruit flies [46]. During field experiment Ladd traps captured maximum number of Oriental fruit flies significantly due to protein odour volatiles [47]. Papaya juice and guava pulp play an important role for the attraction of fruit flies during behavior and physiological olfaction analysis based on these synthetic food baits appealed significantly more attraction towards *Bactrocera* spp. [48]. Guava fruits produce a wide range of volatile compounds i.e., aldehydes, ketones and sesquiterpenes these compounds are responsible for attracting and repelling insects [49, 50].

The Queensland fruit fly (*Bactrocera tryoni* (Froggatt)), a polyphagous pest, was investigated the behavioral olfaction tests in the laboratory. An11-volatile synthetic blend (based on an antenna electrophysiological responses) was formulated based on odours of mature guava (*Psidium guajava*), which had been found to attract female and male fruit flies more strongly than three other ripening stages and guava pulp. The results showed that when guava pulp was mixed with ethyl acetate, ethyl butyrate, and ethyl propionate (pulp + 3Volatiles) a higher proportion of flies were attracted as compared to pulp alone [51].

The behavioral and electrophysiological attraction responses of males and females of the Mexican fruit fly (Diptera: Tephritidae) *Anastrepha ludens* to guava (*Psidium guajava* L.) volatiles were investigated in laboratory tests through wind tunnel tests under controlled condition. Both sexes were more attracted to Porapak Q extracts of guava than to solvent controls. Antennal responses from male and female were detected through Gas chromatography and electro-antenno graphic detection (GC-EAD) analysis of the behaviorally active of fruit flies [52].

Grape juice has recently been used as a bait for monitoring fruit flies, such as *A. fraterculus* [53]. In a field experiment, grape juice (25%), Nulure (5%) and torula yeast extract (2.5%) were used and tested in McPhail traps in a first experiment. A second experiment compared grape juice to Bio *Anastrepha*, a Brazilian produced hydrolyzed protein bait, and *Anastrepha* lure, as well asa third lure of two components (ammonium sulfate and putrescine). In the first experiment, hydrolyzed torula was superior to grape juice or Nulure. In the second experiment, grape juice was almost equivalent to the Bio *Anastrepha* and significantly more attractive than the two components of *Anastrepha* bait [54]. Different baits, including torula yeast extract, grape juice and Biolure, were also evaluated for their attraction of different species of Bactrocera in orchards [55, 56].

A number of experiments has been carried out by various authors using various grape products, and they found that commercial grape juice significantly captured more flies than either torula hydrolysate or Biolure [57]. Numerous grape products were also identified as attractive to Mexican fruit flies in wind tunnels under laboratory conditions [58]. Studies have revealed that combinations of different proteins and food attractant components provided good attraction against different species of fruit flies and were compared with grape juice, powdered grape and torula yeast extract as attractants for Mexican fruit flies. Results indicated that grape juice is superior to powder and at least equal to torula yeast hydrolysate for trapping pest populations of Mexican fruit flies in commercial citrus orchards [35]. Under the field conditions in mango orchard protein-based baits with ammonium acetate and diammonium phosphate showed good attraction to *B. zonata* compared to the mixtures of protein-bait and ammonium compound with Amadene or Agrinal and alone. With respect to Agrinal, when it was mixed with ammonia compounds, it attracted significantly higher numbers of *B. zonata* in comparison to Agrinal alone. On the other hand, Amadene mixed with any of the ammonia compounds more significantly enhanced the attraction of *B. zonata* especially females than males [38].

The efficacy of six ammonium compounds (tri-ammonium phosphate, ammonium carbonate, ammonium acetate, ammonium chloride, ammonium thiocyanate and ammonium dihydrogen phosphate) as baits was evaluate for the adults of *zizyphus* fruit fly, *Carpomya incompleta* (Beeker) under field conditions. Each compound was tested at five concentrations (1%, 2%, 3%, 4%, and 5%). The results indicated that tri-ammonium phosphate, ammonium carbonate and ammonium acetate attracted females more than males. The rest of tested compounds and concentrations attracted both sexes with no significant differences between them [59].

The data also showed variation in the attractiveness of *B. zonata* to different synthetic proteinaceous food baits, such as ammonium acetate, papaya powder + kachri powder, KOH and guava pulp. Results revealed that ammonium acetate attracted the highest number of *B. zonata* at all the tested baits, confirming that it was the most attractive bait for both male and female *B zonata* [60]. The proteinaceous food baits with three synthetic food attractants i.e., putrescence, trimethylamine, ammonium acetate, were found to be more effective in capturing female Mediterranean fruit fly (*C. capitata*) than males in citrus orchard [61]. In addition, protein hydrolysate with ammonium acetate could capture the blueberry maggot *Rhagoletis mendax *(Curran) fruit fly [62]. The synthetic food baits with three component of attractant trimethylamine, putrescence and ammonium acetate were proved to be resourceful attractive to female *C. capitata* [63]. In a laboratory bioassay, the main volatile compounds emitted from hydrolyzed protein and yeast that are attractive to West Indian fruit flies *A. obliqua* Macquart (Diptera: Tephritidae) were identified in a wind tunnel. Yeast is a good attractant itself for *A. oblique* but addition of sugar also stimulates feeding activity of fruit flies [64].

The present study convincingly demonstrates the enhanced response of *B. zonata,* especially in female, to ammonium acetate, which can be added to a verity of protein baits and materials. Different protein baits (Protein hydrolysate, jaggery, guava pulp, papaya powder, kachri powder, KOH) mixed with three attractant components (Ammonium acetate (AA), Trimethylamine (TMA) and Putrescine (Pu)) in various combinations were used for the attraction of *B. zonata* fruit fly in the Y-olfactometer.

## Conclusion

In conclusion, our study found that both sexes of *B. zonata* are attracted to the bait 1, 6, 8 (ammonium acetate based) and bait 6 and bait 8 (guava pulp based) attractants throughout the adult life span (5 days to 30 days old). Bait 4 and bait 2 is exclusively effective on females. Similarly, some baits were used for early detection of mass trapping, some for intermediate position, and some for last stage of fruit fly life span due to physiological cues (Visual, size, sense of touch) and olfactory cues (odours, volatile, nutrition), and attraction response also depends on gravid or non-gravid female *B. zonata*. Ammonium acetate, trimethylamine, and putrescine can be added to baits to increase their potential to attract *B. zonata* flies. Females of *B. zonata* were more sensitive to acidic vs alkaline pH levels than males. Further research on the influence of pH on the efficacy of different attractants in attracting fruit flies in varied ecological settings is required. It is also concluded that three compound ammonium based attractant mores potent as well as two or single based ammonium compounds.

## Data Availability

The datasets analyzed during this study are available from the corresponding author on reasonable request. All the necessary data are presented in the paper.
